# Identifying altered developmental pathways in human globoid cell leukodystrophy iPSCs-derived NSCs using transcriptome profiling

**DOI:** 10.1186/s12864-023-09285-6

**Published:** 2023-04-19

**Authors:** Yafeng Lv, Yu Qin, Jing Wang, Guoshuai Tian, Wei Wang, Chunyu Cao, Ye Zhang

**Affiliations:** 1 Hubei Key Laboratory of Tumor Microenvironment and Immunotherapy, College of Basic Medical Sciences, China Three Gorges University, Yichang, 443000 Hubei China; 2 The People’s Hospital of China Three Gorges University, The First People’s Hospital of Yichang, Yichang, 443000 Hubei China; 3 China-Japan Friendship Hospital, Beijing, 100029 China; 4 State Key Laboratory of Medical Molecular Biology, Department of Biochemistry and Molecular Biology, Institute of Basic Medical Sciences, Chinese Academy of Medical Sciences & Peking Union Medical College, Beijing, 100005 China

**Keywords:** Galactocerebrosidase, Globoid cell leukodystrophy, Induced pluripotent stem cell, Neural stem cell, Neurodegenerative

## Abstract

**Background:**

Globoid cell leukodystrophy (GLD) is a devastating neurodegenerative disease characterized by widespread demyelination caused by galactocerebrosidase defects. Changes in GLD pathogenesis occurring at the molecular level have been poorly studied in human-derived neural cells. Patient-derived induced pluripotent stem cells (iPSCs) are a novel disease model for studying disease mechanisms and allow the generation of patient-derived neuronal cells in a dish.

**Results:**

In this study, we identified gene-expression changes in iPSCs and iPSC-derived neural stem cells (NSCs) from a patient with GLD (K-iPSCs/NSCs) and normal control (AF-iPSCs/NSCs), in order to investigate the potential mechanism underlying GLD pathogenesis. We identified 194 (K-iPSCs vs. AF-iPSCs) and 702 (K-NSCs vs. AF-NSCs) significantly dysregulated mRNAs when comparing the indicated groups. We also identified dozens of Gene Ontology and Kyoto Encyclopedia of Genes and Genomes pathway terms that were enriched for the differentially expressed genes. Among them, 25 differentially expressed genes identified by RNA-sequencing analysis were validated using real-time quantitative polymerase chain reaction analysis. Dozens of pathways involved in neuroactive ligand–receptor interactions, synaptic vesicle cycle signaling, serotonergic synapse signaling, phosphatidylinositol–protein kinase B signaling, and cyclic AMP signaling were identified as potential contributors to GLD pathogenesis.

**Conclusions:**

Our results correspond to the fact that mutations in the galactosylceramidase gene may disrupt the identified signaling pathways during neural development, suggesting that alterations in signaling pathways contribute to GLD pathogenesis. At the same time, our results demonstrates that the model based on K-iPSCs is a novel tool that can be used to study the underlying molecular basis of GLD.

**Supplementary Information:**

The online version contains supplementary material available at 10.1186/s12864-023-09285-6.

## Background

Globoid cell leukodystrophy (GLD), also known as Krabbe disease, is a lysosomal storage disease that is triggered by a deficit of the lysosomal enzyme galactosylceramidase (GALC) and is characterized by psychosine (PSY) accumulation in the nervous system [1]. A total of 140 mutations are known to cause GLD in the human *GALC* gene located on chromosome 14q13 [2–4]. The absence of GALC results in the accumulation of PSY which has strong neurotoxic activity that can lead to extensive demyelination, neuroinflammation, and axonal degeneration of the nervous system [2]. In addition to acting on myelin, PSY affect glial and neuronal cell homeostasis, autophagy, and promote apoptosis and lysosomal dysfunction [5–7]. Growing evidence exists for previously underappreciated associations among lysosomal dysfunction, protein misfolding, lipid imbalances, and aging in multiple organisms and cells [8, 9] which provides a basis for studying these processes as potential contributors to GLD pathology.

Small and large animal models of GLD have been widely used to study primary and secondary pathological events and to test new therapies [10–15]. Although the biochemical and histological features of these animal models are similar to those of human patients with GLD and partially recapitulate the spectrum of pathological manifestations observed in patients, large differences among species make it difficult for animal models to accurately reflect the clinical pathophysiology of humans, which increases the difficulty of extending the efficacy and toxicity of test results of potential drugs from animals to humans [16]. In addition, human cell models of GLD include patient-specific fibroblasts, hematopoietic cells, or epithelial cell lines with *GALC* mutations, which poorly recapitulate the metabolic and functional characteristics of neural cells [10].

In 2006, Professor Yamanaka of Kyoto University (Kyoto, Japan) developed a method to prepare induced pluripotent stem cells (iPSCs) [17] with almost the same pluripotency as embryonic stem cells that can be obtained directly from skin fibroblasts, blood cells, and other somatic cells. Thus, patient-derived iPSCs can provide an unlimited supply of disease-relevant cells in a personalized manner, making them an extremely valuable resource for previously unavailable cell types, such as cardiomyocytes and neurons [18, 19]. Since disease-specific iPSCs were established in 2008, research in this field has continued to expand globally [20–27]. By utilizing these disease-specific iPSCs, researchers can reproduce the pathological changes in a patient’s disease in the laboratory, and disease-specific iPSC-derived neural cells faithfully replicate biological activities in the same patient’s neural cells. Therefore, iPSC technology has great potential for facilitating biomedical research on human nervous system diseases, and iPSCs have become one of the most attractive disease models.

Previously, we isolated fibroblasts from the patient with GLD and normal control and successfully induced their differentiation into iPSCs (K-iPSC from the patient; AF-iPSC from the control) [28, 29]. Here, we differentiated the iPSCs into neural stem cells (NSCs) and established a reliable human neural cell model of GLD. In addition, the gene-expression profiles of K-iPSCs/NSCs compared with AF-iPSCs/NSCs were analyzed to identify differentially expressed genes (DEGs) and gain insight into the potential mechanism of GLD pathogenesis.

## Results

### K-iPSCs showed lower GALC enzyme activities and could be induced to differentiate into NSCs in vitro

**Fig. 1:**
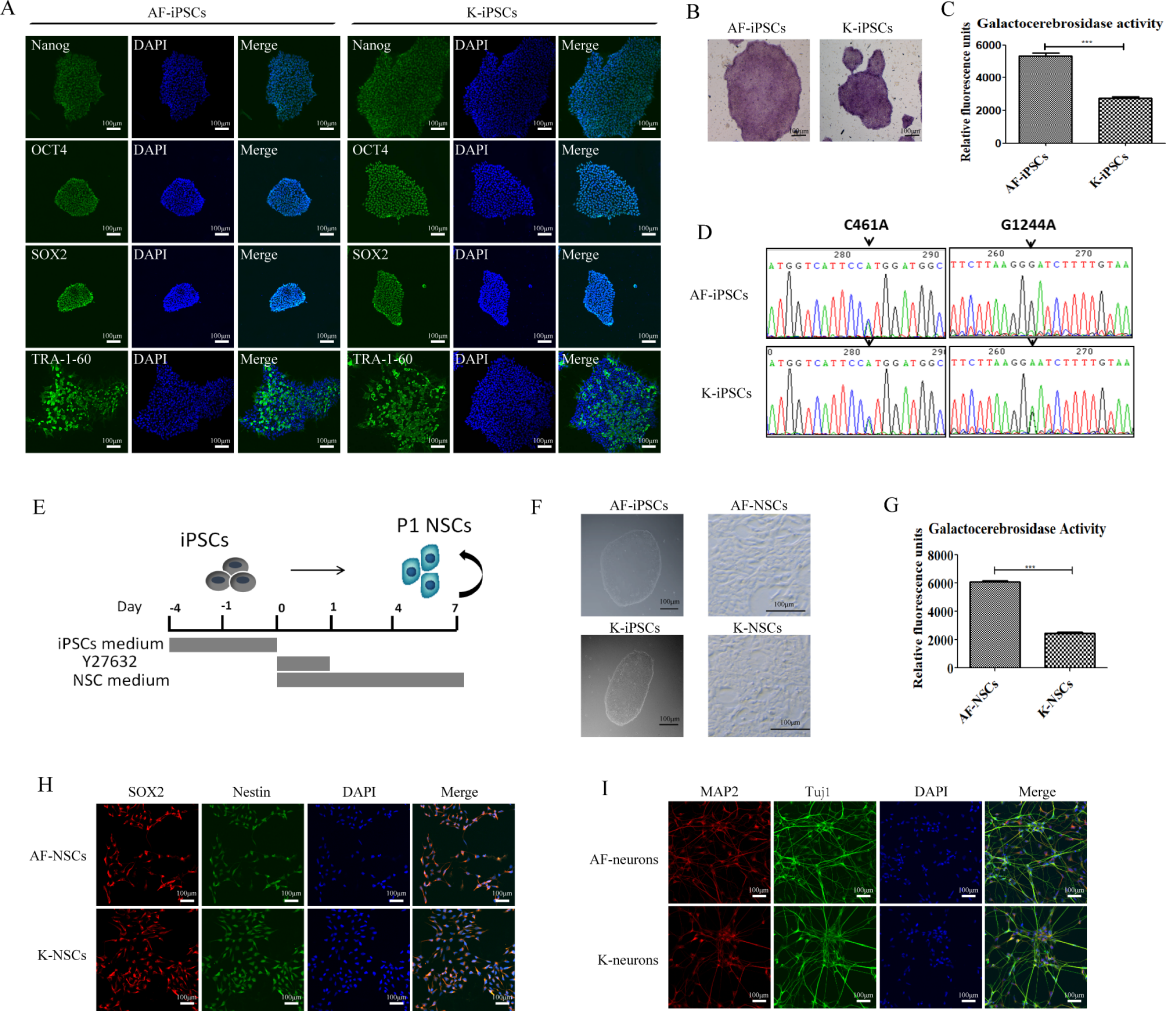
K-iPSCs showed a significant reduction of GALC enzyme activity and could be differentiated into NSCs *in vitro*. **(A)** Immunofluorescence images showing the expression of Nanog, OCT4, SOX2, and TRA-1-60 in the AF-iPSC and K-iPSC lines. Nuclei were counterstained with DAPI (blue). Scale bars, 100 μm. **(B)**Alkaline phosphatase activity of the AF-iPSC and K-iPSC lines. **(C)** GALC enzymatic activity in the AF-iPSC and K-iPSC lines. Student’s *t*-test, n = 4, ***p < 0.001. **(D)***GALC* mutations in the K-iPSC line. Genetic sequencing showed compound heterozygous mutations in the *GALC* gene (c.461 C > A, c.1244G > A). **(E)** Protocol used to differentiate iPSCs into NSCs. **(F)** Representative bright-field images of the iPSC and NSC lines. Scale bars, 100 μm. **(G)** GALC enzymatic activity in the AF-NSC and K-NSC lines. Student’s *t*-test, n = 4, ***p < 0.001. **(H)** Immunofluorescence images showing the expression of Nestin and SOX2 in the AF-NSC and K-NSC lines. Nuclei were counterstained with DAPI (blue). Scale bars, 100 μm. **(I)** Immunofluorescence images showing the expression of MAP2 and Tuj1 in the AF-neuron and K-neuron lines. Nuclei were counterstained with DAPI (blue). Scale bars, 100 μm.

Previously, we used a Sendai virus encoding transcription factors (OCT4, KLF4, c-MYC, and SOX2) to successfully induce fibroblasts from a patient with GLD to differentiate into iPSCs (K-iPSCs) [28]. In this study, iPSCs derived from an asymptomatic father of the patient with GLD (AF-iPSCs) were used as a control. Pluripotency-related genes were expressed in K-iPSCs and AF-iPSCs (Fig. 1A). Alkaline phosphatase activity analysis showed that both K-iPSCs and AF-iPSCs were positive (Fig. 1B). The *GALC* gene of the patient with GLD enrolled in this study had a double heterozygous mutation, c.461 C > A and c.1244G > A. The c.461 C > A mutation came from the patient’s father, and the c.1244G > A mutation came from the patient’s mother. To test whether the *GALC* gene mutation was retained in iPSCs, we extracted genomic DNA from the K-iPSCs and AF-iPSCs, and amplified the DNA near the mutation site by PCR. Sanger sequencing confirmed that the same mutations were present in both types of iPSCs (Fig. 1D). The karyotype of the K-iPSCs and AF-iPSCs were inspected by Giemsa-banding, and the results showed normal diploid 46, XY karyotype, without any detectable abnormalities at 10 passages (Supplementary Fig. 1). GALC enzyme activity is often used for the diagnosis of the GLD disease. In this study, we detected GALC enzyme activity in two iPSC lines. The results showed that the GALC enzyme activity in AF-iPSCs was significantly higher than that in K-iPSCs (Fig. 1C), indicating that GLD disease may result in abnormal GALC enzyme activity during early embryonic development.

NSCs are favorable models for studying the pathogenesis of neurological diseases. Ethical limitations and limited cell sources make it difficult to directly obtain human NSCs from patients. Because the pathological features of GLD mainly comprise abnormalities of the nervous system, we differentiated both iPSC lines into NSCs to establish a neural cell model of GLD. In this study, we used serum-free NSC-induction medium (Life Technologies), which can efficiently differentiate human iPSCs into NSCs within 1 week without the need for embryoid body formation and the mechanical isolation of NSCs. The differentiation process is shown in Fig. 1E. NSCs had a typical NSC morphology (Fig. 1F). Unlike the iPSCs, NSCs showed single cell growth, cell dispersion, and small cell diameter. As in iPSCs, GALC enzyme activity in AF-NSCs was significantly higher than that in K-iNSCs (Fig. 1G ). Immunofluorescence staining was used to analyze the expression of NSC-specific markers(Figure. 1H). We selected Nestin and SOX2 as molecular markers to identify NSCs. Nestin belongs to the intermediate filament protein family and is specifically expressed in embryonic and NSCs, but not in mature neural cells, and is widely used as a biomarker for NSCs. Sox2, a transcription factor, plays an important role in the maintenance of stemness. In addition, NSCs were differentiated into neurons to confirm that they are indeed generating NSCs from iPSCs (Fig. 1I). There was no significant difference in the expression levels of Nestin and SOX2 between K-NSCs and AF-NSCs, and both can be differentiated into neurons, indicating that K-iPSCs and AF-iPSCs have been successfully induced into NSCs.

### Early transcriptional changes in K-iPSCs and K-NSCs revealed by next-generation high-throughput RNA-seq

**Fig. 2:**
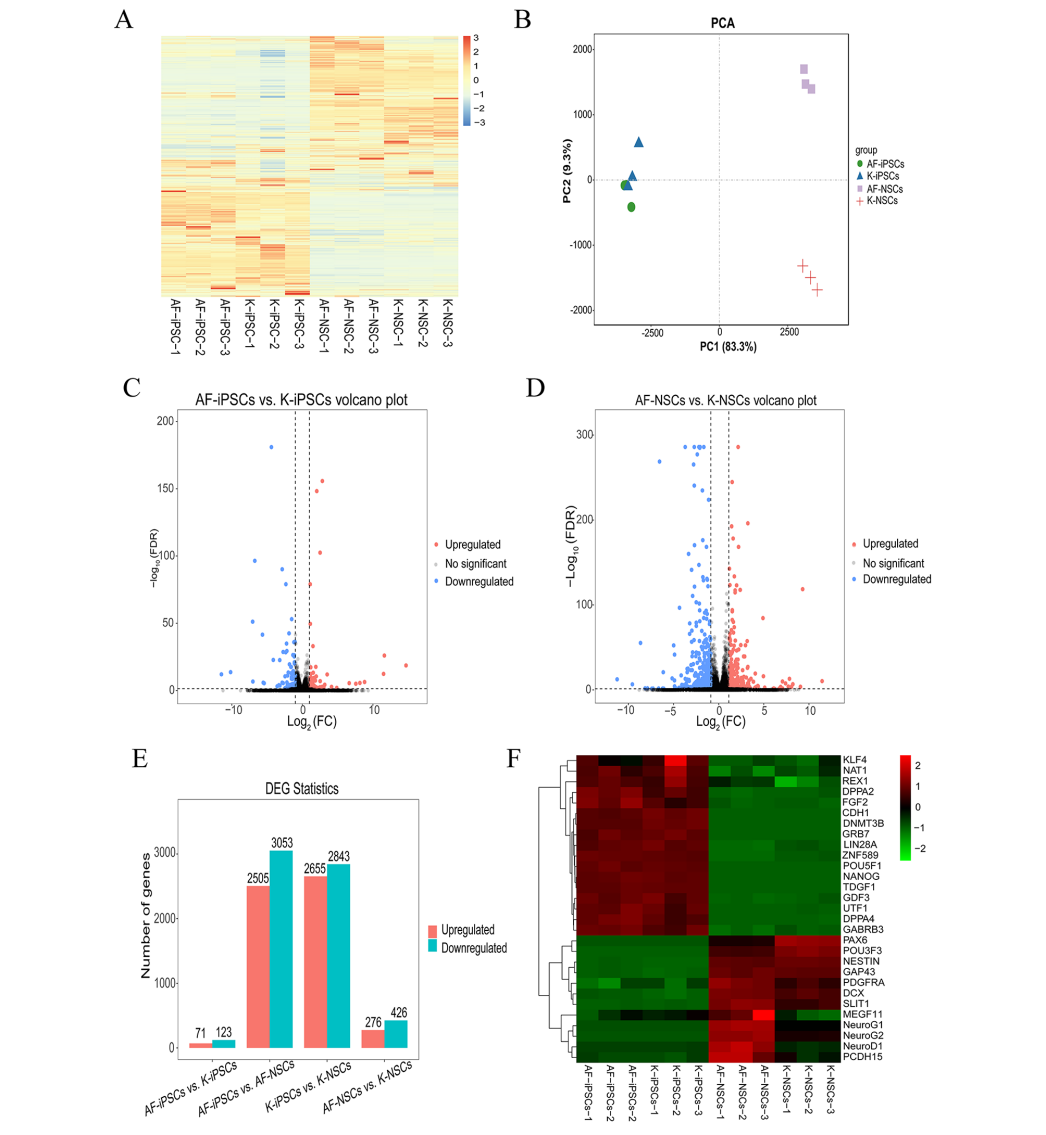
RNA-seq analysis of the iPSC and NSC lines showed early transcriptional changes. **(A)** Heat map representing the gene-expression patterns of DEGs found when comparing the K-iPSC and K-NSC lines to control AF-iPSC and AF-NSC lines, using an adjusted p-value of < 0.05 and baseMean cutoff of > 50. Red shading represents elevated expression and blue shading represents decreased expression. **(B)** Principal component analysis of global gene-expression values. **(C–D)** Volcano plot-based differential expression analysis of significantly downregulated or upregulated mRNAs in K-iPSCs vs. AF-iPSCs, or K-NSCs vs. AF-NSCs. **(E)** Histogram displaying the number of upregulated and downregulated genes. **(F)** Heat map representing the expression of candidate marker genes in iPSC and NSC lines. Red shading represents upregulated expression and green shading represent downregulated expression. The figure labelling AF-iPSCs-1,2,3, K-iPSCs-1,2,3, AF-NSCs-1,2,3 and K-NSCs-1,2,3 represent the technical replicates.

Four groups of iPSCs (AF-iPSCs, K-iPSCs, AF-NSCs, and K-NSCs) were used for transcriptome sequencing, and each group of samples was analyzed in three replicates. Data filtering was performed before data analysis to reduce any analysis interference caused by invalid data. The proportion of clean reads in all groups exceeded 99.8%, and comparison with the reference genome was as high as 96%. When analyzing the RNA-seq data, we generated statistical values for all four comparison groups: AF-iPSCs vs. K-iPSCs, AF-NSCs vs. K-NSCs, AF-iPSCs vs. AF-NSCs, and K-iPSCs vs. K-NSCs. Heat map diagrams, PCA graphs, and volcano plots were generated for the gene-expression analysis (Fig. 2A-D).

We set the FDR and FC cut-off values to create lists of significantly dysregulated mRNAs in each comparison group. Transcripts with an FDR value of < 0.05 and a |log_2_ FC| value of > 1 were considered to be differentially expressed. We counted the DEGs in each group. In the first group, 194 significantly dysregulated mRNAs were identified by comparing the K-iPSC lines with the AF-iPSC line (123 downregulated genes and 71 upregulated genes). After differentiation into NSCs, the number of DEGs between the AF-NSC and K-NSC groups reached 702, including 276 upregulated genes and 426 downregulated genes. The number of transcripts that were differentially expressed in both the iPSC and NSC lines is shown in the histogram diagram (Fig. 2E). Lists of the 30 most dysregulated transcripts in the iPSC and NSC groups are shown in Supplementary Tables S3 and S4.

Next, we analyzed the expression levels of some iPSC and NSC biomarkers. The results are expressed in the form of a heat map (Fig. 2F). The K-iPSC and AF-iPSC lines specifically expressed iPSC marker genes, and the K-NSC and AF-NSC lines specifically expressed NSC marker genes. These results further verified the established iPSC and NSC lines at the transcriptome level, and the obtained iPSC and NSC lines had typical gene-expression signatures. In addition, after iPSCs were induced into NSCs, the expression of some genes, such as *MEGF11*, *NeuroG1*, *NeuroG2*, *NeuroD1*, and *PCDH15*, were significantly lower in K-NSCs than in AF-NSCs. The reason for the differential expression in the NSC line may be related to the mutation of *GALC* in K-NSCs, which may reflect a pathological mechanism underlying GLD.

**Fig. 3:**
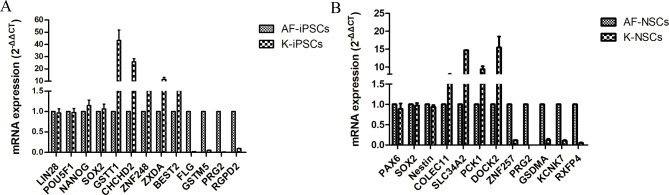
Validation of the expression levels of 25 randomly selected genes by RT-qPCR. **(A)** The 13 mRNAs selected for expression analysis in iPSCs. **(B)** The 12 mRNAs selected for expression analysis in NSCs. GAPDH gene expression was detected as an internal control and relative FC of gene expression found for each gene was calculated using the comparative 2^−ΔΔCT^ method. The RT-qPCR data are presented as the mean ± standard deviation.

To confirm the accuracy and reproducibility of the transcriptome results, 25 mRNAs were selected to verify the RNA-seq data via qPCR. We selected 13 mRNAs from the iPSC lines (*LIN28*, *POU5F1*, *NANOG*, *SOX2*, *GSTT1*, *CHCHD2*, *ZNF248*, *ZXDA*, *BEST2*, *FLG*, *GSTM5*, *PRG2*, and *RGPD2*) and 12 mRNAs (*PAX6*, *SOX2*, *NESTIN*, *COLEC11*, *SLC34A2*, *PCK1*, *DOCK2*, *ZNF257*, *PRG2*, *GSDMA*, *KCNK7*, and *RXFP4*) from the NSC lines. The qPCR results were completely consistent with the upregulation or downregulation observed in the RNA-seq data. These findings suggest that the RNA-seq data served as a reliable readout for differential expression lines (Fig. 3).

### GO analysis of down-regulated genes in iPSCs and NSCs

**Fig. 4:**
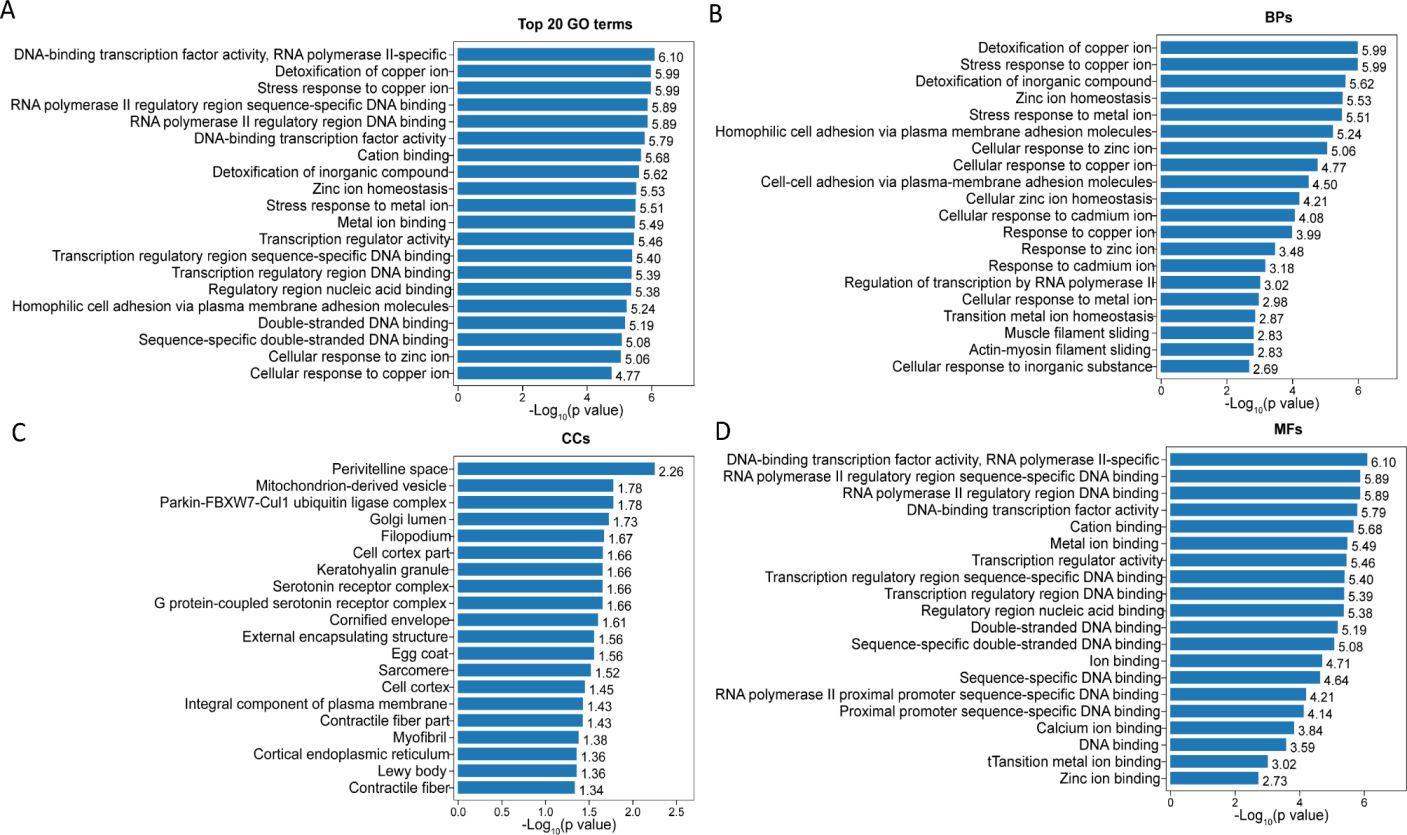
Analysis of significantly enriched GO terms between AF-iPSCs and K-iPSCs. **(A)** The top 20 GO terms found in the enrichment analysis. **(B)** The top 20 BP terms found in the enrichment analysis. **(C)** The top 20 CC terms found in the enrichment analysis. **(D)** The top 20 MF terms found in the enrichment analysis.

To further characterize the downregulated genes related to *GALC* mutations in the iPSC and NSC groups, GO enrichment analysis was performed using ClusterProfiler. With iPSCs, enrichment analysis revealed 123 downregulated genes that were significantly (p < 0.05) enriched for 2,530 GO terms, where 1963 were related to BPs, 284 were related to CCs, and 283 were related to MFs. The top five most enriched BP terms were detoxification of Cu ions (GO:0010273), stress response to Cu ions (GO:1,990,169), detoxification of inorganic compounds (GO:0061687), Zn ion homeostasis (GO:0006882), and stress response to metal ions (GO:0097501). DNA-binding transcription factor activity (GO:0003700), RNA polymerase II regulatory region sequence-specific DNA binding (GO:0000977), RNA polymerase II regulatory region DNA binding (GO:0001012), DNA-binding transcription factor activity (GO:0000981), and cation binding (GO:0043169) were the top five enriched MF terms. The top five enriched CC terms were perivitelline space (GO:0098595), mitochondria-derived vesicle (GO:0099075), Parkin-FBXW7-Cul1 ubiquitin ligase (GO:1,990,452), Golgi lumen (GO:0005796), and filopodium (GO:0046847). Figure 4 shows the top 20 GO terms related to the downregulated genes between K-iPSCs and AF-iPSCs, in terms of their enrichment factors.

**Fig. 5:**
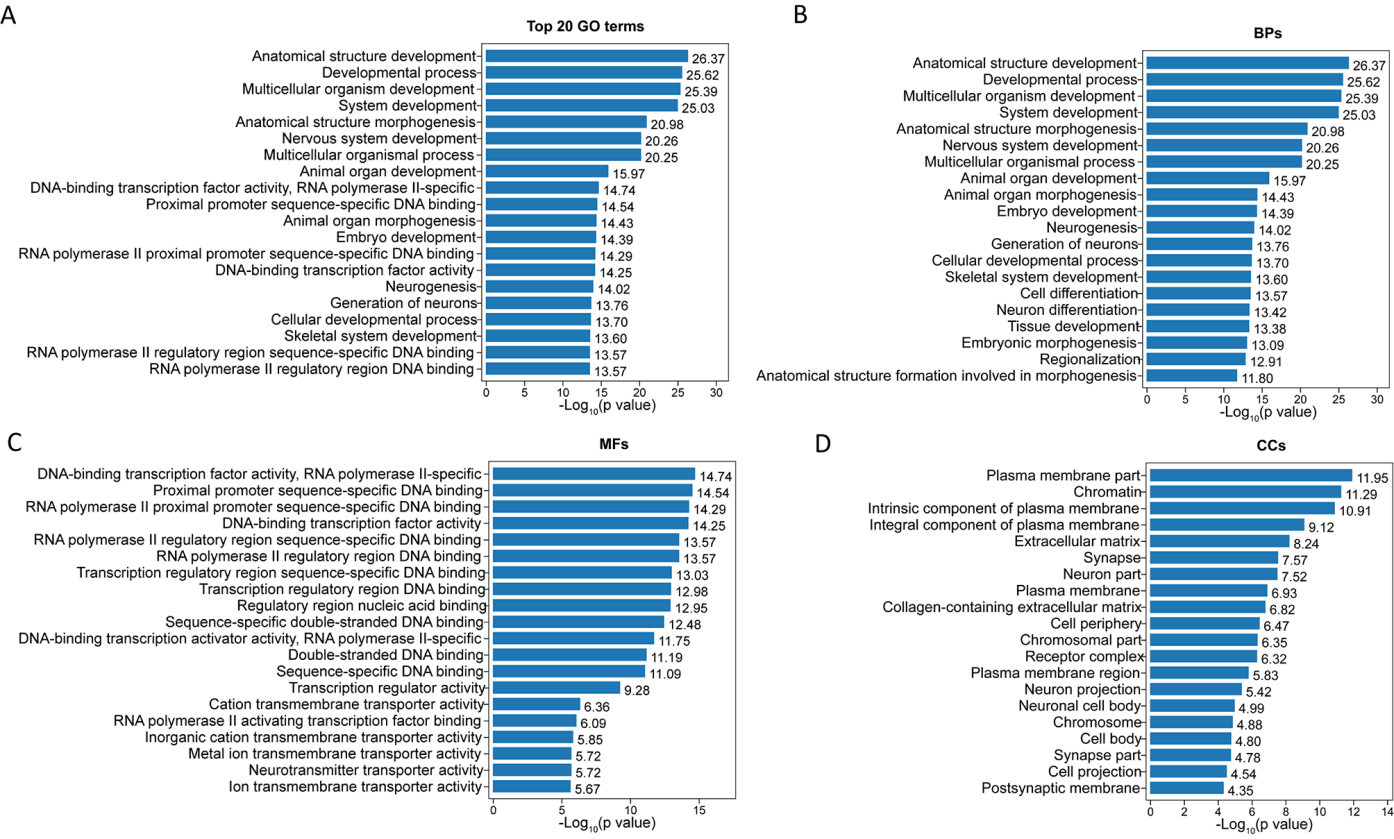
Analysis of significantly enriched GO terms between AF-NSCs and K-NSCs. **(A)** The top 20 GO terms found in the enrichment analysis. **(B)** The top 20 BP terms found in the enrichment analysis. **(C)** The top 20 CC terms found in the enrichment analysis. **(D)** The top 20 MF terms found in the enrichment analysis.

After the K-iPSCs and AF-iPSCs differentiated into NSCs, the number of downregulated genes increased to 426. The downregulated genes in both groups of NSCs were subjected to GO functional-enrichment analysis. All 426 downregulated genes in NSCs were significantly (p < 0.05) enriched for GO terms (n = 6,927), including 5,493 BP, 567 CC, and 867 MF terms. The top five enriched BP terms were anatomical structural development (GO:0048856), developmental process (GO:0032502), multicellular organism development (GO:0007275), system development (GO:0002520), and anatomical structural morphogenesis (GO:0022603). Moreover, we found that many of the significantly enriched terms were related to nervous system development, including single-organism developmental processes (GO:0044767), nervous system development (GO:0007399), neurogenesis (GO:0022008), neuron differentiation (GO:0030182), and central nervous system development (GO:0007417). DNA-binding transcription factor activity (GO:0003700), proximal promoter sequence-specific DNA binding (GO:0000978), RNA polymerase II proximal promoter sequence-specific DNA binding (GO:0000978), DNA-binding transcription factor activity (GO:0048513), and RNA polymerase II regulatory region sequence-specific DNA binding (GO:0000977) were the top five enriched MF terms. The five most enriched CC terms were plasma membrane part (GO:0044459), chromatin (GO:0006325), intrinsic component of the plasma membrane (GO:0031226), integral component of the plasma membrane (GO:0005887), and extracellular matrix (GO:0044420). Figure 5 shows the top 20 GO terms related to downregulated genes, in terms of the enrichment factors.

### KEGG pathway analysis of down-regulated genes in iPSCs and NSCs

**Fig. 6:**
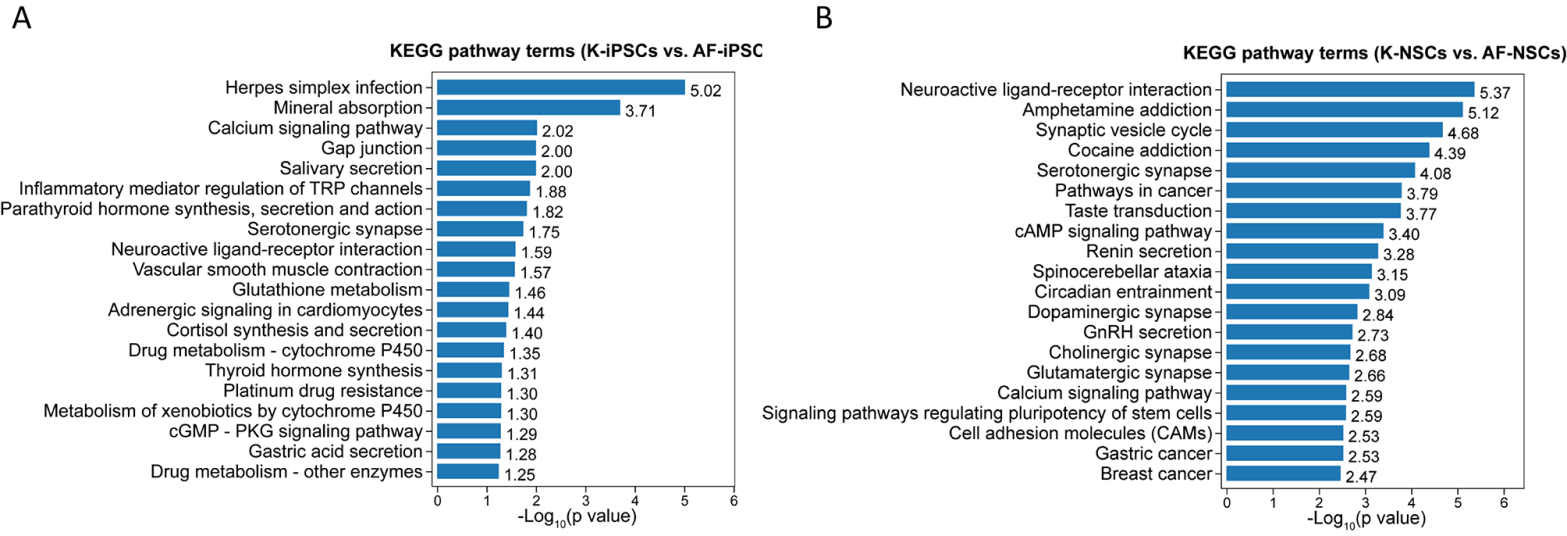
The top 20 enriched KEGG pathway terms associated with DEGs between different comparison groups. **(A)** K-iPSCs vs. AF-iPSCs. **(B)** K-NSCs vs. AF-NSCs. KEGG analysis was performed based on the method as described by Kanehisa et al [50].

In organisms, different genes perform their biological functions in coordination. Pathway-based analysis helps further understand the biological functions of genes. The KEGG Database is the main public database used for pathway analysis. Pathway enrichment can determine the most important biochemical, metabolic, and signal-transduction pathways associated with different genes. We performed KEGG functional enrichment analysis of the downregulated genes in AF-iPSCs and K-iPSCs. The 123 downregulated genes were enriched for 107 pathway terms, including 16 significant terms (p < 0.05). The top five enriched pathways were mineral absorption (ko04978); gap junction (ko04540); calcium signaling pathway (ko04020); salivary secretion (ko04970); and parathyroid hormone synthesis, secretion, and action (ko04928). Figure 6 A shows the top 20 KEGG pathway terms related to the downregulated genes, according to their FDR values.

We identified 426 downregulated genes in K-NSCs and AF-NSCs, which were mainly enriched for 221 pathway terms, including 47 terms with a p-value of < 0.05. The top five enriched pathways were neuroactive ligand-receptor interaction (ko04080), amphetamine addiction (ko05031), synaptic vesicle cycle (ko04721), cocaine addiction (ko05030), and serotonergic synapses (ko04726). Moreover, we found that many signaling pathways have been reported in previous studies, such as the PI3K-Akt signaling pathway (ko04151), cAMP signaling pathway (ko0415), and MAPK signaling pathway (ko04010). Figure 6B shows the top 20 KEGG pathway terms related to the downregulated genes, according to their FDR values.

## Discussion

GLD is a neurodegenerative disorder caused by GALC activity. Clinical symptoms include irritability, difficulty swallowing, progressive spasms, cognitive impairment, blindness, deafness, and seizures. Most patients develop symptoms in the first 6 months of life, although some patients develop symptoms later in life or even in adulthood. Even patients with the same genotype may have different clinical manifestations and pathological progressions, and the exact pathological mechanism of the disease is currently unknown. At present, the most commonly used model to study GLD is the twitcher mouse [30]. The twitcher mouse is a naturally occurring mutant that contains a premature stop codon (W339X) in the *GALC* gene, which results in complete loss of enzymatic activity. The pathophysiology of the homozygous twitcher mouse model are similar to those of human GLD patients in many aspects, including central and peripheral nerve demyelination, weight loss, hind limb ataxia, kyphosis, and severe tremor [30, 31]. However, there are still many differences in brain structure and gene expression between species make the twitcher mouse unable to fully summarize the clinical pathophysiological processes of humans. iPSCs not only have the ability of self-renewal and multi-directional differentiation, but can also be directly obtained from skin fibroblasts, blood cells, and other somatic cells from patients. Thus, patient-derived iPSCs can provide an unlimited supply of disease-relevant cells in a personalized manner. This is an extremely valuable resource for previously unavailable cell types such as cardiomyocytes and neurons.

In this study, we differentiated K-iPSCs derived from a patient with GLD into NSCs established an NSC model for studying the pathogenesis of GLD. Nestin, SOX2 were selected as molecular markers to identify NSCs via immunofluorescence staining. Positive expression of Nestin and SOX2 were detected in K-NSCs and AF-NSCs. In order to confirm that they are indeed generating NSCs from iPSCs, NSC was differentiated into neurons expressed MAP2 and Tuj1, which indicating that the iPSCs were successfully differentiated into NSCs. Both cell lines from the patient with GLD patient and the patient’s father were successfully induced into NSCs.

In this study, the transcriptomes of iPSCs and NSCs were sequenced using the Illumina HiSeq2500 platform. Statistical analysis revealed 194 DEGs between the AF-iPSC and K-iPSC groups, including 71 upregulated genes and 123 downregulated genes. After inducing differentiation into NSCs, 702 DEGs, including 276 upregulated and 426 downregulated genes, were identified between the AF-NSC and K-NSC groups. After the iPSCs differentiated into NSCs, the number of DEGs between both groups increased significantly. In addition, several genes (*KLF4*, *NAT1*, *REX1*, *DPPA2*, *FGF2*, *CDH1*, *DNMT3B*, *GRB7*, *LIN28A*, *ZNF589*, *POU5F1*, *NANOG*, *TDGF1*, *GDF3*, *UTF1*, *DPPA4*, and *GABRB3*) specifically expressed by iPSC were highly expressed in K-iPSCs and AF-iPSCs, but poorly expressed in K-NSCs and AF-NSCs. We also identified several genes (*PAX6*, *POU3F3*, *VIM*, *DCX*, *NESTIN*, *GAP43*, *PDGFRA*, *SLIT1*, *NCAM1*) specifically expressed by NSCs that were expressed at low levels in K-iPSCs and AF-iPSC, but were highly expressed in AF-NSCs and K-NSCs. Our results further verified the successful establishment of the iPSC and NSC lines at the transcriptome level. It should be noted that some genes (such as *MEGF11*, *NeuroG1*, *NeuroG2*, *NeuroD1*, and *PCDH15*) were highly expressed in AF-NSCs, but poorly expressed in K-NSCs. Previously, *MEGF11* was shown implicated in retinal development, and the typical clinical symptom of GLD observed was vision loss [32]. *NeuroG1*, *NeuroG2*, and *NeuroD1* are neural-specific basic transcription factors that can specify the neuronal fate in ectodermal cells and are expressed in neural progenitor cells within the developing central and peripheral nervous systems. In addition, we observed some downregulated genes (such as *MPZ*, *GJB1*, *CDH15*, *KCNQ3*, and *PLP1*) in K-NSC cells, and abnormal expression of these genes has been reported to cause cognitive impairment, epilepsy, spasticity, and myelin abnormalities, which are similar to the disease symptoms of GLD [33–36]. We suspect that the differential expression of such genes related to neural differentiation in the AF-NSC and K-NSC groups was related to mutations in the *GALC* gene, but further studies are needed to verify this possibility.

GLD pathogenesis has been proposed to arise from the accumulation of PSY at high levels in the central nervous system (CNS) of patients with GLD than in healthy individuals [37, 38]. PSY accumulation leads to cytotoxic effects on oligodendroglial cells and neural progenitors, thereby triggering apoptosis, cell senescence, and alterations in the cellular membrane architecture [39–41]. Additionally, PSY affects various enzymes involved in signal-transduction pathways [42]. In this study, we used previously established iPSC lines established from a patient with GLD as a reliable human model to gain insight into GLD pathogenesis. To this end, we differentiated K-iPSCs into NSCs and performed transcriptome sequencing. Significantly enriched DEG-related pathways between the K-NSCs and AF-NSC lines were identified, including the MAPK signaling pathway, PI3K-Akt signaling pathway, cAMP signaling pathway, and RAS signaling pathway, as well as neuroactive ligand–receptor interactions. The roles of the MAPK signaling pathway, PI3K-Akt signaling pathway, and cAMP signaling pathways in GLD pathology have been reported [43, 44]. The MAPK signaling pathway is a key signaling pathway in cell proliferation, differentiation, apoptosis, and stress responses under normal and pathological conditions. The PI3K/Akt signaling pathway is well known to regulate cell cycle progression and to play important roles in regulating cell division, differentiation, survival, and tumorigenesis. Increasing evidence has shown that the PI3K/Akt signaling pathway also plays various important roles in the CNS, as abnormalities in the signaling pathway are closely related to many diseases of the CNS [45–47]. Insulin-like growth factor I (IGF-1) is a growth factor that activates the anti-apoptotic PI3K-Akt or MAPK signal-transduction pathways [43]. IGF-1 prevented PSY-mediated Akt and Erk1/2 dephosphorylation, which was reversed by inhibitors of the MAPK and PI3K-Akt pathways [43]. T-cell associated gene 8 (TDAG8) is an orphan G-protein-coupled receptor, and PSY is a specific ligand for TDAG8. When a myelomonocytic cell line was exposed to galactosylceramide and PSY, only the latter induced the formation of large multinuclear cells that resembled globoid cells. In PSY-treated cells, the signals that inhibited cytokinesis were transmitted through TDAG8, acting through an increase in cAMP. Therefore, cAMP is an important mediator of PSY-induced multinuclear cell death [44, 48]. Consistent with the above research, we also observed that the MAPK signaling pathway, PI3K-Akt signaling pathway, and cAMP signaling pathway were associated with GLD pathogenesis, based on transcriptome sequencing analysis. In addition, neuroactive ligand–receptor interactions and the cAMP signaling pathway were found to be potentially involved in GLD pathogenesis.

In summary, our results suggest that these signaling pathways are closely related to *GALC* mutations. Investigating the enriched signaling pathways identified here will help understand GLD pathogenesis, identify targets for drug development, and contribute to the prevention and treatment of GLD. It should be noted that since it is not feasible to obtain more GLD iPSCs, all the data in this article are based on a single iPSC clone. Future studies will focus on validating these hypotheses using more iPSC lines derived from patients with GLD and animal models of GLD.

## Conclusion

In this study, a new NSC model of GLD was established that showed good prospects for the fields of disease-mechanism research and drug screening. After sequencing the transcriptomes of iPSC and iPSC-differentiated NSCs, we conducted GO and KEGG pathway enrichment analysis of downregulated genes. Our findings preliminarily revealed the pathological mechanism of GLD disease caused by *GALC* mutations at the transcriptional level. However, further experimental studies are required to verify the roles of the identified genes and pathways.

## Materials and methods

### Inducing human iPSC differentiate into NSCs

We started with high-quality human iPSCs cultured under feeder-free conditions, such as in Essential 8 ™ Medium on vitronectin. When the iPSCs reached ~ 70–80% confluency, they are dislodged to generate cell clumps. The iPSCs were induced into NSCs using the PSC neural induction medium provided by Thermo Fisher Scientific (Catalog Number A1647801) according to the manufacturer instruction. Briefly, NSC culture medium was added to vitronectin (Catalog Number A14700, Thermo Fisher Scientific) coated 6-well plates. Conical tubes containing iPSC suspensions were gently shaken, and then the iPSCs were seeded into wells at a density of 2.5 × 10^5^ iPSCs per well. The plates were moved in several quick back-and-forth and side-to-side motions to disperse the cells across the surface and then gently placed in a CO_2_ incubator. On days 2 and 4, remove any non-neural differentiated colonies and add 5 mL of pre-warmed complete neural induction medium into each well. On day 6 of neural induction, the cells reached near maximal confluence. On day 7 of neural induction, the NSCs (P0) were harvested and expanded. Of note, after dissociating the NSCs, overnight treatment with the ROCK inhibitor Y27632 was required at the time of plating to prevent cell death during both expansion and differentiation.

### Alkaline phosphatase (AP) assay

iPSCs were cultured in a 6-wells plate coated with Geltrex™ (Thermo Fisher Scientific). After 5 days of culture. The clones can be stained with a Millipore kit (SCR004) following the manufacturer’s instructions. The cells were visualized under an inverted fluorescence microscope.

### Immunofluorescence analysis

When conducting immunofluorescence analysis, the culture medium was discarded, the cells were washed twice with phosphate-buffered saline (PBS), fixed with 4% paraformaldehyde for 15 min at room temperature, washed three times with PBS (3 min/wash step), treated with 0.5% TritonX-100 at room temperature for 10 min, and blocked with 3% bovine serum albumin (BSA) at room temperature for 1 h. Each primary antibody was diluted 3% BSA antibody, and an appropriate amount of primary antibody was dropped onto each slide, and the cells were incubated overnight at 4 °C. Subsequently, the cells were washed three times with PBS (3 min/wash step) and incubated for 1 h at room temperature with an appropriate secondary antibody (diluted in PBS). Next, the secondary antibody was discarded, and the cell slides were washed three times with PBS. The nuclei were stained with 4′,6-diamidino-2-phenylindole (DAPI). The details of all the antibodies are listed in Table S1.

### iPSC-derived NSCs differentiate into neurons

The NSCs were induced into neurons using the Gibco™ CultureOne™ Supplement kit provided by Thermo Fisher Scientific (Catalog Number A33202-01) according to the manufacturer instruction. Briefly, before neurons induction, the NSCs were in good condition without any differentiation. The NSCs were digested by StemPro TM Accutase™ (Catalog Number A1110501, Thermo Fisher Scientific) and suspended by DPBS. The NSCs were transferred to a 15 mL centrifuge tube, centrifuged at 800 rpm for 5 min, and the supernatant was discarded. The NSCs were resuspended in 2 mL neuron differentiation medium, and 5 × 10^5^ cells were spread in a 6-well plates coated with Poly-D-Lysine and laminin. Move the culture plates in several quick back-and-forth and side-to-side motions to disperse NSCs across the surface and place them gently in a 37 °C CO_2_ incubator, and the neuron differentiation medium was replaced once every 2–3 days. After 2 weeks, the differentiated neurons could be used to detect.

### GALC activity assay

Specific GALC activity was measured using the Lysosomal Galactocerebrosidase (GALC) Analysis Kit (catalog number M2774, MarkerGene™), per the manufacturer’s recommended protocol. Briefly, the cells were harvested, each culture supernatant was carefully removed, and the cells were lysed in lysis buffer A. The resulting lysates were centrifuged at 27,000×*g* for 30 min, the supernatants were recovered by aspirating them from the tubes, and the volume of lysate containing 50 µg of protein was determined for each sample. Next, 50 µg of total protein from each sample was transferred into different substrate tubes, and the total volume was adjusted to 100 µL with reaction buffer. After mixing the contents of the substrate tubes, the tubes were incubated at 37 °C for 2 h in the dark. The reactions were terminated immediately in stop buffer, the terminated reaction mixtures were transferred to separate opaque 96-well plate. The fluorescence was detected in a on a fluorescence microplate reader, using an excitation wavelength of 365 nm and an emission wavelength of 454 nm.

### Sanger sequencing

Genomic DNA was extracted from iPSCs using an *EasyPure* Genomic DNA Kit (TransGen Biotech). Specific primers were designed using Primer Premier 5.0 software. The frameshift mutation in GALC was amplified using specific primers and analyzed by Sanger sequencing. The sequences of the primers used are shown in Supplementary Table S2.

### RNA extraction, library construction, and sequencing

RNA extraction and library construction were performed as previously described by Lin M [49]. Briefly, total RNA was extracted using the TRIzol Reagent Kit (Invitrogen, Carlsbad, CA, USA) according to the manufacturer’s protocol. RNA quality was assessed using an Agilent 2100 Bioanalyzer (Agilent Technologies, Palo Alto, CA, USA) and verified by RNase-free agarose gel electrophoresis. After total RNA was extracted, eukaryotic mRNA was enriched using Oligo(dT) beads, whereas prokaryotic mRNA was enriched by removing rRNA using the Ribo-Zero™ Magnetic Kit (Epicenter, Madison, WI, USA). The enriched mRNAs were then fragmented into short fragments using fragmentation buffer and reverse transcribed into complementary DNA (cDNA) with random primers. Second-strand cDNA was synthesized using DNA polymerase I, RNase H, dNTP. Then, the cDNA fragments were purified using the QiaQuick PCR Extraction Kit (Qiagen, Venlo, The Netherlands), end-repaired, modified by poly(A) addition, and ligated to Illumina sequencing adapters. The ligation products were size-selected by agarose gel electrophoresis, amplified by the polymerase chain reaction (PCR), and sequenced at Gene Denovo Biotechnology Co. (Guangzhou, China) using an Illumina HiSeq2500 instrument.

### Statistical analysis of the RNA-sequencing (RNA-seq) data

To obtain high-quality clean reads, the reads were filtered using fastp software (version 0.18.0). During the filtering process, we removed reads that (1) containing adapter sequences, (2) containing more than 10% unknown nucleotides, and (3) had an overall low with more than 50% low-quality (Q-value ≤ 20) bases. The resulting paired-end clean reads were mapped to the reference genome using HISAT2 software (version 2.4). The reads for each sample were mapped with StringTie software (version 1.3.1), using a reference-based approach. For each transcribed region, fragments-per-kilobase million values were calculated to quantify the relatives abundances and variations. Differential expression analysis of RNAs between two groups was performed using DESeq2 software. DEGs/transcripts were defined as transcripts with a false-discovery rate (FDR) of < 0.05 and an absolute fold-change (FC) of ≥ 2.

### Gene ontology (GO)-based enrichment analysis

The GO Database covers three ontologies, namely molecular functions (MFs), cellular components (CCs), and biological processes (BPs). The basic unit of the GO Database is the GO term. Each GO term is associated with a particular type of ontology. Gene numbers were calculated for every term, and significantly enriched GO terms for downregulated genes comparing to the genome backgroundwere defined by a hypergeometric test. The calculated p-values were subjected to FDR corrections, with an FDR of ≤ 0.05 serving as the threshold for statistical significance. GO terms meeting this criterion were defined as significantly enriched GO terms among the downregulated genes. This analysis enabled identification of the main biological functions associated with the downregulated genes.

### Pathway-enrichment analysis

The Kyoto Encyclopedia of Genes and Genomes (KEGG) Database is a major public pathway-related database. Pathway-enrichment analysis identified significantly enriched metabolic pathways or signal-transduction pathways associated with downregulated genes, when compared to the whole genome background. Each calculated p-value was subjected to FDR correction, with an FDR of ≤ 0.05 serving as the threshold for statistical significance. Pathways meeting this criterion were defined as significantly enriched pathways associated with the downregulated genes.

Validation of gene expression by quantitative real time polymerase chain reactionTo verify the accuracy of the RNA-seq data, 25 DEGs were randomly selected and analyzed by real-time quantitative PCR. The culture medium for the iPSCs and NSCs was discarded, after which 1 mL of the TRIzol reagent was added, and the resulting mixture was immediately pipetted up and down six to eight times. Next, 0.2 mL of chloroform was added, the tubes were inverted vigorously up and down for 30 s, and then centrifuge at 12,000 rpm and 4 °C for 15 min. For each sample, the upper aqueous phase was transferred to a new 1.5 mL Eppendorf tube. Then, 0.6 mL isopropanol was added, the tubes were centrifuged at 12,000 rpm and 4 °C for 10 min, and the supernatants were discarded. The residual contents were washed once with 1 mL of 75% ethanol, centrifuged at 12,000 rpm and 4 °C for 10 min, the supernatant was discarded, and the pellet was dissolved in 50 µL of RNase-free water after drying.

cDNA was prepared from purified RNA samples using the PrimeScript™ RT Reagent Kit with gDNA Eraser (Takara, Dalian, China). Specific primers were designed using Primer Premier 5.0 according to human gene sequences deposited in GenBank. The corresponding primer sequences are shown in Supplementary Table S2. Human β-actin was detected as the internal reference gene, and primers were synthesized by GENEWIZ Biotechnology Co., Ltd. cDNA was used as the template, diluted 5–10-fold for the experiments, and amplified on a Bio-Rad Fluorescence Quantitative PCR instrument. At least three replicate wells were used for each sample. The qPCR conditions were as follows: 5 min of initial denaturation at 95 °C; 40 three-step cycles of denaturation at 94 °C for 30 s, annealing at 58 °C for 30 s, and elongation at 72 °C for 20 s. The qPCR analysis was performed using a CFX96 instrument. The relative expression level of each gene was calculated using the comparative threshold cycle (2^−ΔΔCt^) method, using the human β-actin gene as the internal reference. SPSS software (version 11.5) was used for the statistical analysis.

## Electronic supplementary material

Below is the link to the electronic supplementary material.

**Additional file 1: Supplementary Table S1.** Antibodies used for immunocytochemistry.

**Additional file 2: Supplementary Table S2.** Primers used i real time quantitative PCR and sanger sequencing.

**Additional file 3: Supplementary Table S3.** List of 30 the most dysregulated mRNAs in K-iPSC lines compared to AF-iPSC lines.

**Additional file 4: Supplementary Table S4.** List of 30 the most dysregulated mRNAs in K-NSC lines compared to AF-NSC lines.

**Additional file 5:** The karyotype of the K-iPSCs and AF-iPSCs were inspected by Giemsa-banding, and the results showed normal diploid 46, XY karyotype, without any detectable abnormalities at 10 passages.

## Data Availability

All the RNA-seq data were uploaded to the GEO repository (accession number GSE212512 ) https://www.ncbi.nlm.nih.gov/geo/query/acc.cgi?acc=GSE212512.

## Abbreviations

AF-iPSC: iPSC derived from the asymptomatic father of a patient with GLD
AF-NSC: NSC differentiated from an AF-iPSC
BP: biological process
BSA: bovine serum albumin
CC: cellular component
cDNA: complementary DNA
CNS: central nervous system
DAPI: 4’,6-diamidino-2-phenylindole
FC: fold-change
FDR: false-discovery rate
GALC: galactosylceramidase
GLD: globoid cell leukodystrophy
GO: Gene Ontology
iPSC: induced pluripotent stem cell
K-iPSC: iPSC reprogramed from fibroblasts of GLD patient
K-NSC: NSC differentiated from a K-iPSC
KEGG: Kyoto Encyclopedia of Genes and Genomes
MF: molecular function
NSC: neural stem cell
PBS: phosphate-buffered saline
PCR: polymerase chain reaction
PSY: psychosine
RT-qPCR: reverse transcriptase-quantitative polymerase chain reaction
