# Supplementary material for: Identifying altered developmental pathways in human globoid cell leukodystrophy iPSCs-derived NSCs using transcriptome profiling
Source: BMC Genomics. 2023 Apr 19;24:210. doi: 10.1186/s12864-023-09285-6 (PMC10116706; doi:10.1186/s12864-023-09285-6)
Supplement: Supplementary file 1 — Additional file 1: Supplementary Table S1. Antibodies used for immunocytochemistry. [file 12864_2023_9285_MOESM1_ESM.docx]

Supplementary Table S1. Antibodies used for immunocytochemistry

| Antibody | Dilution | Company Cat |
| --- | --- | --- |
| Rabbit anti-SOX2 | 1:200 | Millipore (Cat#AB5603) |
| Rabbit anti-OCT4 | 1:200 | Cell Signaling Technology(Cat#2750) |
| Rabbit anti-NANOG | 1:200 | Cell Signaling Technology(Cat#3580) |
| Anti-TRA-1–60 | 1:200 | Thermo Fisher Scientific(Cat#A25618) |
| Mouse anti-Nestin | 1:200 | Millipore (Cat# MAB5326) |
| Anti-Rabbit Alexa Fluor 594 | 1:200 | Thermo Fisher Scientific(Cat# A11012) |
| Anti-Mouse Alexa Fluor 488 | 1:200 | Thermo Fisher Scientific(Cat# A11029) |
