# Supplementary material for: Identifying altered developmental pathways in human globoid cell leukodystrophy iPSCs-derived NSCs using transcriptome profiling
Source: BMC Genomics. 2023 Apr 19;24:210. doi: 10.1186/s12864-023-09285-6 (PMC10116706; doi:10.1186/s12864-023-09285-6)
Supplement: Supplementary file 2 — Additional file 2: Supplementary Table S2. Primers used i real time quantitative PCR and sanger sequencing. [file 12864_2023_9285_MOESM2_ESM.docx]

|  | Gene | Forward primer | Reverse primer |
| --- | --- | --- | --- |
| RT-qPCR | GAPDH | GTCTCCTCTGACTTCAACAGCG | ACCACCCTGTTGCTGTAGCCAA |
|  | GSTT1 | AGCACACGACTCTGCGGAGAAG | AGGGTCACATCCAACTCTGCCA |
|  | CHCHD2 | GTGGAGGAAGTAATGCTGAGCC | CACAGAGCTTGATGTCACCCTG |
|  | PNMA6A | CCAGGTGTTTGGAGACAACGAG | CACTTCCAGCCGCAACACGAAA |
|  | FLG | GCTGAAGGAACTTCTGGAAAAGG | GTTGTGGTCTATATCCAAGTGATC |
|  | ZNF248 | TGCTGGACCCTGCTCAGAAGAT | TATCCAGGGCTCTTCTCCTTGC |
|  | ZXDA | CTGGGCACAAAGCCTTTCGTGT | GCAACGGCTTTTCCAAGTGTCC |
|  | GSTM5 | CACATGGAGCTGGTCAGACTGT | CTTGTCTCCTGCAAACCATGGC |
|  | PRG2 | CCTGGTGAGAAGTCTTCAGACG | CGCTGACAGAACACTGGATTCG |
|  | BEST2 | TAGCTGAGCAGCTCATCAACCC | ACATCTCGTCCACTGCCAGCAT |
|  | RGPD2 | GGAAACAGAGGCAACCAGTGCA | CTTCAAGTGTTCCACGTTAGCTG |
|  | LIN28 | CCAGTGGATGTCTTTGTGCACC | GTGACACGGATGGATTCCAGAC |
|  | POU5F1 | CCTGAAGCAGAAGAGGATCACC | AAAGCGGCAGATGGTCGTTTGG |
|  | NANOG | CTCCAACATCCTGAACCTCAGC | CGTCACACCATTGCTATTCTTCG |
|  | SOX2 | GCTACAGCATGATGCAGGACCA | TCTGCGAGCTGGTCATGGAGTT |
|  | PAX6 | CTGAGGAATCAGAGAAGACAGGC | ATGGAGCCAGATGTGAAGGAGG |
|  | NESTIN | TCAAGATGTCCCTCAGCCTGGA | AAGCTGAGGGAAGTCTTGGAGC |
|  | ZNF257 | GATCACCTGTCTGGAGCAAGGA | ATGTCTCGCTCTGGGCAAAGGT |
|  | COLEC11 | GATGGACAACCAGGTCTCTCAG | GCTTCTCCTCCTTCACCAGCAG |
|  | GSDMA | AGAACAGCACTCTGGAGGTCCA | CCATCACCACATACAGGTTCTCC |
|  | KCNK7 | GCCGTCTACTTCTGCTTCAGCT | CCAAGAGTCCTAGAAGCAAGTAAC |
|  | SLC34A2 | GACCTACAAGGAGAACATCGCC | ATCAGGCAACCACAGAGGACCA |
|  | RXFP4 | CCTGTCACTACTTGCTTGGCAC | TCAACCGCAGATCCCTGAAGGT |
|  | PCK1 | CATTGCCTGGATGAAGTTTGACG | GGGTTGGTCTTCACTGAAGTCC |
|  | DOCK2 | TGAAGCTGGACCACGAGGTAGA | GCCTTTGACCAGGTTCACGAAG |
| Genotyping | GALC (461C > A) | CTATTGGTAAGGGTCTTGGA | GGTATACACTCAACACAGTT |
|  | GALC (1244G > A | GCCATTGACCTTTGTAT | GCTTGAAAGTTCCCTAG |

Supplementary Table S2. Primers used i real time quantitative PCR and sanger sequencing
