# Supplementary material for: Identifying altered developmental pathways in human globoid cell leukodystrophy iPSCs-derived NSCs using transcriptome profiling
Source: BMC Genomics. 2023 Apr 19;24:210. doi: 10.1186/s12864-023-09285-6 (PMC10116706; doi:10.1186/s12864-023-09285-6)
Supplement: Supplementary file 3 — Additional file 3: Supplementary Table S3. List of 30 the most dysregulated mRNAs in K-iPSC lines compared to AF-iPSC lines. [file 12864_2023_9285_MOESM3_ESM.docx]

Supplementary Table S3 | List of 30 the most dysregulated mRNAs in K-iPSC lines compared to AF-iPSC lines

| Symbol | Ensembl gene | | Log2FoldChange | PValue | FDR |
| --- | --- | --- | --- | --- | --- |
| *GSTT1* | ENSG00000184674 | 14.63159392 | | 1.04E-21 | 3.34E-19 |
| *CHCHD2* | ENSG00000106153 | | 11.56071495 | 2.79E-29 | 1.47E-26 |
| *FLG* | ENSG00000143631 | | -10.22881869 | 1.37E-16 | 6.19E-13 |
| *ZNF248* | ENSG00000198105 | | 8.759333407 | 1.40E-08 | 3.14E-14 |
| *ZNF662* | ENSG00000182983 | | 8.094517599 | 8.50E-08 | 6.98E-07 |
| *ZXDA* | ENSG00000198205 | | 7.518325308 | 4.12E-07 | 3.44E-06 |
| *AC079062.1* | ENSG00000263711 | | -7.101037699 | 6.66E-55 | 1.33E-05 |
| *GSTM5* | ENSG00000134201 | | -7.069673528 | 5.88E-09 | 9.47E-52 |
| *PRG2* | ENSG00000186652 | | -6.797787832 | 1.76E-100 | 3.10E-07 |
| *BEST2* | ENSG00000039987 | | 6.579944426 | 1.30E-07 | 5.02E-97 |
| *RGPD2* | ENSG00000185304 | | -5.710493383 | 4.24E-45 | 4.87E-06 |
| *GOLGA6L18* | ENSG00000215749 | | -5.599912842 | 4.72E-08 | 4.30E-42 |
| *GOLGA6L19* | ENSG00000259243 | | -5.473415848 | 1.56E-07 | 2.04E-06 |
| *PNMA6D* | ENSG00000257088 | | 4.818759685 | 2.31E-03 | 5.67E-06 |
| *NNAT* | ENSG00000053438 | | -4.446898119 | 9.73E-186 | 0.01521997 |
| *DDX43* | ENSG00000080007 | | -4.179004295 | 5.70E-26 | 1.38E-181 |
| *GATSL1* | ENSG00000183086 | | 4.076102979 | 2.52E-04 | 2.70E-23 |
| *AC069368.1* | ENSG00000249240 | | -3.625834782 | 8.53E-05 | 0.002714877 |
| *LRRC61* | ENSG00000127399 | | 3.456514135 | 1.02E-14 | 0.001122363 |
| *ANKRD1* | ENSG00000148677 | | -3.380142709 | 7.81E-06 | 1.65E-12 |
| *GSTT2* | ENSG00000099984 | | -3.264157563 | 7.85E-26 | 0.000160296 |
| *AC073610.2* | ENSG00000272822 | | -3.247927513 | 1.86E-05 | 3.49E-23 |
| *HLA-DQB1* | ENSG00000179344 | | 3.113341473 | 3.06E-06 | 0.000324137 |
| *A2M* | ENSG00000175899 | | 2.979822118 | 7.22E-04 | 7.30E-05 |
| *OOEP* | ENSG00000203907 | | -2.918295359 | 4.64E-08 | 0.006165287 |
| *ZNF729* | ENSG00000196350 | | -2.917660118 | 4.11E-94 | 2.04E-06 |
| *CCDC169* | ENSG00000250709 | | -2.783188611 | 1.21E-07 | 9.73E-91 |
| *PCDH19* | ENSG00000165194 | | -2.780218792 | 4.80E-32 | 4.61E-06 |
| *INPP5F* | ENSG00000198825 | | 2.774105418 | 3.19E-160 | 3.10E-29 |
| *ALG10B* | ENSG00000175548 | | 2.754174884 | 2.22E-04 | 2.27E-156 |
