# Supplementary material for: Identifying altered developmental pathways in human globoid cell leukodystrophy iPSCs-derived NSCs using transcriptome profiling
Source: BMC Genomics. 2023 Apr 19;24:210. doi: 10.1186/s12864-023-09285-6 (PMC10116706; doi:10.1186/s12864-023-09285-6)
Supplement: Supplementary file 4 — Additional file 4: Supplementary Table S4. List of 30 the most dysregulated mRNAs in K-NSC lines compared to AF-NSC lines. [file 12864_2023_9285_MOESM4_ESM.docx]

Supplementary Table S4 | List of 30 the most dysregulated mRNAs in K-NSC lines compared to AF-NSC lines

| Symbol | Ensembl gene | Log2FoldChange | PValue | FDR |
| --- | --- | --- | --- | --- |
| *ZNF257* | ENSG00000197134 | -9.736965594 | 3.08E-08 | 4.15E-07 |
| *CHCHD2* | ENSG00000106153 | 9.131734496 | 1.00E-121 | 4.36E-119 |
| *COLEC11* | ENSG00000118004 | 8.886712714 | 3.86E-05 | 0.000305857 |
| *PRG2* | ENSG00000186652 | -8.830737021 | 5.32E-58 | 8.02E-56 |
| *GSDMA* | ENSG00000167914 | -8.321928095 | 0.004940415 | 0.021017816 |
| *KCNK7* | ENSG00000173338 | -8.058893689 | 0.004811101 | 0.02057885 |
| *ZXDA* | ENSG00000198205 | 8.040746342 | 2.15E-08 | 3.00E-07 |
| [*CYP4F31P*](http://feb2014.archive.ensembl.org/Homo_sapiens/Gene/Summary?g=ENSG00000178206&db=core) | ENSG00000178206 | 7.886712714 | 4.59E-05 | 0.000358012 |
| [*GSTT1*](http://feb2014.archive.ensembl.org/Homo_sapiens/Gene/Summary?g=ENSG00000184674&db=core) | ENSG00000184674 | 7.671098726 | 5.01E-15 | 1.38E-13 |
| *C5orf58* | ENSG00000234511 | -7.569855608 | 0.012443999 | 0.045612019 |
| *SLC34A2* | ENSG00000157765 | 7.544320516 | 1.88E-05 | 0.000158875 |
| *RXFP4* | ENSG00000173080 | -7.437405312 | 0.009924053 | 0.037728747 |
| *COL8A1* | ENSG00000144810 | 7.238404739 | 2.15E-09 | 3.42E-08 |
| *PCK1* | ENSG00000124253 | 6.906890596 | 0.007658546 | 0.030470089 |
| *ZNF248* | ENSG00000198105 | 6.894817763 | 9.55E-12 | 1.98E-10 |
| *ETNPPL* | ENSG00000164089 | -6.824428435 | 0.007249178 | 0.029130636 |
| *NNAT* | ENSG00000053438 | -6.724935024 | 1.50E-272 | 2.54E-269 |
| *DOCK2* | ENSG00000134516 | 6.691161905 | 5.98E-05 | 0.000452208 |
| *GBP2* | ENSG00000162645 | 6.64385619 | 0.009287205 | 0.035696835 |
| *CHRNA9* | ENSG00000174343 | 6.594946589 | 0.008559714 | 0.033424839 |
| *EVX1* | ENSG00000106038 | 6.544320516 | 0.007232197 | 0.029088833 |
| [*HNF1B*](http://feb2014.archive.ensembl.org/Homo_sapiens/Gene/Summary?g=ENSG00000108753&db=core) | ENSG00000108753 | -6.380821784 | 0.008535398 | 0.033360306 |
| *TBX2* | ENSG00000121068 | -6.312882955 | 5.41E-23 | 2.42E-21 |
| *CD22* | ENSG00000012124 | -6.26052755 | 0.010434823 | 0.039356108 |
| *ADGRF5* | ENSG00000069122 | 5.984893108 | 0.010901224 | 0.040862146 |
| [*ANKRD20A3*](http://feb2014.archive.ensembl.org/Homo_sapiens/Gene/Summary?g=ENSG00000132498&db=core) | ENSG00000132498 | 5.906890596 | 0.0079621 | 0.031447715 |
| *TLX2* | ENSG00000115297 | -5.327466278 | 1.57E-15 | 4.50E-14 |
| *FOSB* | ENSG00000125740 | -5.157403081 | 4.62E-55 | 6.33E-53 |
| *NXPH1* | ENSG00000122584 | -5.055282436 | 9.10E-06 | 8.26E-05 |
