# Supplementary material for: Identifying altered developmental pathways in human globoid cell leukodystrophy iPSCs-derived NSCs using transcriptome profiling
Source: BMC Genomics. 2023 Apr 19;24:210. doi: 10.1186/s12864-023-09285-6 (PMC10116706; doi:10.1186/s12864-023-09285-6)
Supplement: Supplementary file 5 — Additional file 5: The karyotype of the K-iPSCs and AF-iPSCs were inspected by Giemsa-banding, and the results showed normal diploid 46, XY karyotype, without any detectable abnormalities at 10 passages. [file 12864_2023_9285_MOESM5_ESM.docx]

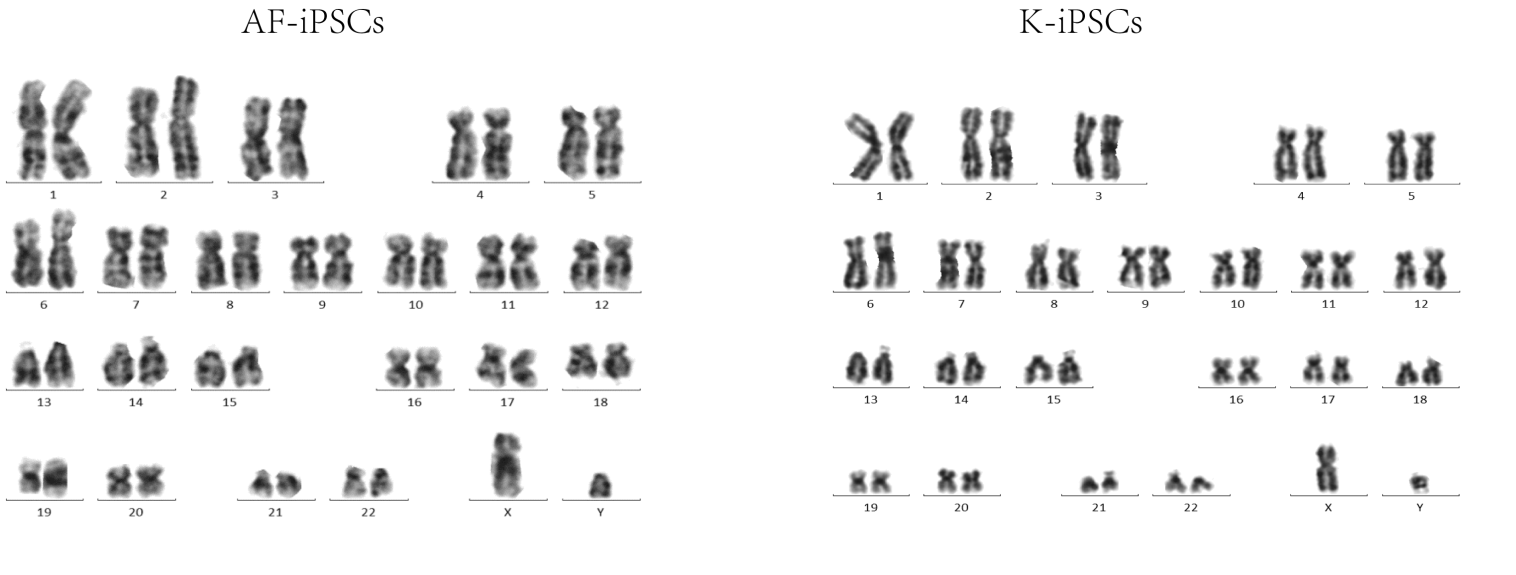
The karyotype of the K-iPSCs and AF-iPSCs were inspected by Giemsa-banding, and the results showed normal diploid 46, XY karyotype, without any detectable abnormalities at 10 passages. Fifteen metaphases were counted.
